# Overproduction of active efflux pump and variations of OprD dominate in imipenem-resistant *Pseudomonas aeruginosa* isolated from patients with bloodstream infections in Taiwan

**DOI:** 10.1186/s12866-016-0719-2

**Published:** 2016-06-13

**Authors:** Cheng-Yen Kao, Shu-Sheng Chen, Kuei-Hsiang Hung, Hsiu-Mei Wu, Po-Ren Hsueh, Jing-Jou Yan, Jiunn-Jong Wu

**Affiliations:** Department of Medical Laboratory Science and Biotechnology, College of Medicine, National Cheng Kung University, Tainan, Taiwan; Department of Microbiology and Immunology, College of Medicine, National Cheng Kung University, Tainan, Taiwan; Department of Internal Medicine, National Taiwan University Hospital, College of Medicine, National Taiwan University, Taipei, Taiwan; Department of Pathology, National Cheng Kung University Hospital, College of Medicine, National Cheng Kung University, Tainan, Taiwan; Department of Biotechnology and Laboratory Science in Medicine, School of Biomedical Science and Engineering, National Yang-Ming University, Taipei, Taiwan

**Keywords:** Imipenem, *Pseudomonas aeruginosa*, β-lactamase, Efflux pump, OprD

## Abstract

**Background:**

The emergence of imipenem-resistant *Pseudomonas aeruginosa* (IRPA) has become a great concern worldwide. The aim of this study was to investigate resistance mechanisms associated with bloodstream isolated IRPA strains in Taiwan.

**Results:**

A total of 78 non-duplicated IRPA isolates were isolated from patients with bloodstream infection. The average prevalence of imipenem-resistance in those isolates was 5.9 % during a 10-year longitudinal surveillance in Taiwan. PFGE results showed high clonal diversity among the 78 isolates. VIM-2, VIM-3, OXA-10, and OXA-17 β-lactamases were identified in 2 (2.6 %), 3 (3.8 %), 2 (2.6 %), and 1 (1.3 %) isolates, respectively. Active efflux pumps, AmpC β-lactamase overproduction, and extended-spectrum AmpC cephalosporinases (ESACs) were found in 58 (74.4 %), 25 (32.1 %) and 15 (19.2 %) of IRPA isolates, respectively. *oprD* mutations with amino acid substitution, shortened putative loop L7, premature stop codon caused by point mutation, frameshift by nucleotide insertion or deletion, and interruption by insertion sequence were found in 19 (24.4 %), 18 (23.1 %), 15 (19.2 %), 14 (17.9 %), and 10 (12.8 %) of isolates, respectively.

**Conclusions:**

This study suggests that alterations in the OprD protein and having an active efflux pump are the main mechanisms associated with bloodstream isolated IRPA. Overproduction of AmpC, ESACs, and the presence of VIM- and OXA-type β-lactamases play additional roles in reduced susceptibility to imipenem in *P. aeruginosa* isolates in Taiwan.

**Electronic supplementary material:**

The online version of this article (doi:10.1186/s12866-016-0719-2) contains supplementary material, which is available to authorized users.

## Background

*Pseudomonas aeruginosa* is one of the most important nosocomial pathogens, which colonizes patients with trauma or a breach by tracheostomy, catheters, surgery, or severe burns. It is also the major cause of chronic lung infections in individuals with cystic fibrosis [1]. Treatment of infections caused by *P. aeruginosa* has become more challenging because of its resistance to many antimicrobial agents [2, 3]. Carbapenems (including imipenem, meropenem, and doripenem) remain the principal antimicrobial agents for treatment of serious infections, such as nosocomial pneumonia, serious nosocomial intra-abdominal infection and septicemia caused by *P. aeruginosa* [4].

Extensive use of carbapenems has contributed to the emergence of carbapenem-resistant *P. aeruginosa* (CRPA), and the increasing incidence of resistance to carbapenems in *P. aeruginosa* has become a global issue [5–10]. Surveillance of antimicrobial susceptibility by the European Centre for Disease Prevention and Control reported that the average rate of resistance of *P. aeruginosa* against carbapenems was 17.1 % [11].

The acquisition of carbapenem-hydrolyzing β-lactamases (such as KPC, AIM, DIM, GIM, IMP, NDM, SPM, VIM, and OXA), overexpression of chromosome-encoded AmpC β-lactamase, extended-spectrum AmpC cephalosporinases (ESACs), reduction of permeability of the outer membrane protein OprD, and overexpression of the major resistance-nodulation-division (RND) efflux pump systems (MexAB-OprM, MexCD-OprJ, MexEF-OprN, and MexXY-OprM), are all involved in carbapenem resistance in *P. aeruginosa* [2, 12, 13]. Most AmpC-type β-lactamases naturally produced by Gram-negative organisms hydrolyse amino- and ureido-penicillins, cephamycins (cefoxitin and cefotetan) and, to a lesser extent, oxyimino-cephalosporins (such as ceftazidime) and monobactams [14]. In contrast, ESACs confer reduced susceptibility to cephalosporins and carbapenems [15, 16].

The present study was conducted to investigate the prevalence and characteristics of bloodstream isolated imipenem-resistant *P. aeruginosa* (IRPA) collected between 2000 and 2010. Our findings demonstrated that the prevalence of imipenem resistance in bacteremic *P. aeruginosa* remains low (5.9 %) in Taiwan. Mutations in *oprD* and production of an active efflux pump are the major causes of imipenem resistance in *P. aeruginosa*, followed by overproduction of AmpC β-lactamase, ESACs, and the acquisition of VIM and OXA.

## Methods

### Sampling and isolation of *P. aeruginosa*

Bacteremic *P. aeruginosa* isolates were recovered in two Taiwanese University hospitals, January 2000 to February 2010. The Ethics Committee of National Cheng Kung University Hospital (NCKUH) approved that no formal ethical approval was needed to use these clinically obtained materials, because the strains were remnant from patient samples, and the data were analyzed anonymously. *P. aeruginosa* was identified in clinical laboratory by colony morphology, Gram stain, biochemical tests, and the Vitek system (bioMérieux, Marcy l’Etoile, France) according to the manufacturer’s recommendations. Susceptibility to imipenem for *P. aeruginosa* isolates was determined by the disk diffusion method (imipenem, 10 μg/disc) on Mueller-Hinton (MH) agar based on the CLSI guidelines [17]. A total of 78 bacteremic imipenem-resistant *P. aeruginosa* isolates were selected for further analysis.

### Antimicrobial susceptibility testing

Minimal inhibitory concentrations (MICs) of amikacin (Sigma-Aldrich, St. Louis, MO), aztreonam (Sigma), cefepime (Sigma), ceftazidime (Sigma), ciprofloxacin (Sigma), doripenem (Sigma), gentamicin (Amresco Inc., Solon, OH), imipenem (Sigma), levofloxacin (Sigma), and meropenem (Sumitomo Pharmaceuticals Co., Ltd., Osaka, Japan) were determined in duplicate by the agar dilution method according to the recommendations of the CLSI [17]. *P. aeruginosa* ATCC 27853 was used as the quality control strain. The interpretation of resistance to these antimicrobial agents was determined according to the recommendations of the CLSI [18]. Multidrug resistant (MDR) *P. aeruginosa* was defined as isolates that were resistant to at least 3 classes of the tested antimicrobial agents [19].

### Pulsed-field gel electrophoresis (PFGE)

PFGE of *Spe*I-digested genomic DNA samples of *P. aeruginosa* isolates was carried out with a CHEF Mapper XA apparatus (Bio-Rad Laboratories, Hercules, CA) according to the instruction manual. Electrophoresis was performed for 24 h at 14 °C with pulse time ranging from 5 to 35 s at 6 V/cm [20]. PFGE profiles were analyzed and compared using the GelCompar II software, version 2.0 (Unimed Healthcare Inc., Houston, TX).

### β-lactamase characterization

β-lactamase genes (*bla*_CTX-M_, *bla*_GES_, *bla*_GIM_, *bla*_IMP_, *bla*_KPC_, *bla*_OXA-1_, *bla*_OXA-2_, *bla*_OXA-23_, *bla*_OXA-40_, *bla*_OXA-48_, *bla*_OXA-51_, *bla*_OXA-58_, *bla*_PSE_, *bla*_SHV_, *bla*_SIM_, *bla*_SPM_, *bla*_TEM_, and *bla*_VIM_) were detected by PCR [21–30]. The purified PCR products were directly sequenced using an automated ABI PRISM 3730 DNA sequencer (Applied Biosystems, Foster, CA). The DNA sequences and deduced amino acid sequences were compared with genes in the GenBank database (http://www.ncbi.nlm.nih.gov/genbank/) or the β-lactamase classification system (http://www.lahey.org/studies/) to confirm the subtypes of β-lactamase genes.

AmpC overproduction was investigated using cloxacillin (250 μg/mL; Sigma)-containing plates, since cloxacillin inhibits AmpC β-lactamase activity and thus restores susceptibility to ceftazidime [16]. *E. cloacae* ATCC 13047, which contained AmpC class C β-lactamase, and ATCC 15337 were used as positive and negative control strains, respectively [30]. ESACs β-lactamases properties were determined by using cloxacillin-containing plates with or without adding imipenem [16]. An isolate was defined as an AmpC overproducer or having ESACs phenotype when there was at least a twofold dilution difference between the MIC of ceftazidime/ceftazidime plus cloxacillin and the MIC of imipenem/imipenem plus cloxacillin, respectively. In addition, PCR amplification of *ampC*-type genes was performed as previously described [16]. The purified PCR products were directly sequenced using an automated ABI PRISM 3730 DNA sequencer (Applied Biosystems, Foster, CA). AmpC sequences were compared with that of *P. aeruginosa* PAO1 strain (GenBank nucleotide sequence accession numbers FJ666065).

### Detection of efflux pump activity

To investigate the activity of efflux pumps in IRPA isolates, MICs of meropenem in the presence of efflux pump inhibitors Phe-Arg β-naphthylamide dihydrochloride (PAβN) (Sigma) or carbonyl-cyanide-*m*-chlorophenylhydrazone (CCCP) (Sigma) were determined [12, 31, 32]. PAβN at 50 μg/mL or 12.5 μM CCCP was incorporated in MH agar, and meropenem susceptibility testing was performed in parallel on agar plates with or without an efflux pump inhibitor. *P. aeruginosa* ATCC 27853 was used as the control strain.

### Analysis of efflux pump genes expression

Overnight cultured bacteria were diluted 1:100 in MH broth and grown to the late log phase of growth (OD_600_ ~ 0.7). Total RNA was extracted using the acid phenol/chloroform method and treated with RNase-free DNase I (Promega, Madison, WI) and RNasin (Promega), according to a previous study [33]. RT-qPCR was performed and the expression of the housekeeping *proC* gene was used as the internal control for relative quantification [34]. Oligonucleotide primers to examine the gene expression of *mexA*, *mexC*, *mexE*, *mexX*, and *proC* were described previously [13, 34]. Isolates were considered to be MexAB-OprM, MexCD-OprJ, MexEF-OprN, and MexXY-OprM hyperproducers when the levels of expression of *mexA*, *mexC*, *mexE*, and *mexX* were at least fivefold higher than that of the reference strain *P. aeruginosa* PAO1, respectively.

### Examination of *oprD* mutations

The *oprD* gene of *P. aeruginosa* was amplified by PCR, and the primers used were described previously [6]. *oprD* sequences were compared with that of the *P. aeruginosa* PAO1 strain. Insertion sequences (ISs) were further identified using the IS Finder database (http://www-is.biotoul.fr) [35].

## Results

### Long-term surveillance and antimicrobial susceptibility of IRPA

Based on the large scale screening of bacteremic *P. aeruginosa* isolates, 5.9 % of isolates appeared to be resistant to imipenem. The trend in the prevalence of imipenem-resistant invasive isolates remained generally stable and low during this 10-year surveillance. The MICs of the 78 isolates to 10 antimicrobial agents are shown in Table 1. All isolates were resistant to imipenem but 2 (2.6 %) and 3 (3.8 %) isolates were susceptible to meropenem and doripenem, respectively. Moreover, the entire collection was highly susceptible to amikacin (67/78, 85.9 %). No colistin resistant IRPA isolate was found. A total of 65 (83.3 %) IRPA isolates were defined to be MDR strains.

**Table 1:** In vitro activity of 10 antimicrobial agents against 78 bloodstream infection IRPA isolates

Antibiotic^a^	MIC (μg/mL)			% Susceptibility		
	Range	MIC_50_	MIC_90_	S	I	R
Cephems						
Ceftazidime	2–> 128	32	128	29.5	14.1	56.4
Cefepime	2–> 64	32	64	21.8	16.7	61.5
Monobactam						
Aztreonam	2–> 64	32	64	24.4	21.8	53.8
Carbapenems						
Imipenem	16–256	32	64	0	0	100
Meropenem	1–64	16	32	2.6	9.0	88.4
Doripenem	1–128	16	32	3.8	0	96.2
Aminoglycosides						
Amikacin	≤1–> 64	8	32	85.9	5.1	9.0
Gentamicin	≤1–> 256	8	256	33.3	23.1	43.6
Fluoroquinolones						
Ciprofloxacin	≤0.5–> 64	2	32	47.4	5.1	47.5
Levofloxacin	≤0.5–> 128	4	64	42.3	9.0	48.7

MIC_50/90_, minimum inhibitory concentration for 50 % and 90 % of the isolates, respectively; S, susceptible; I, intermediate resistant; R, resistant

^a^All isolates were susceptible to colisin, as determined by the disc diffusion method

### Pulsed-field gel electrophoresis analysis

**Fig. 1:**
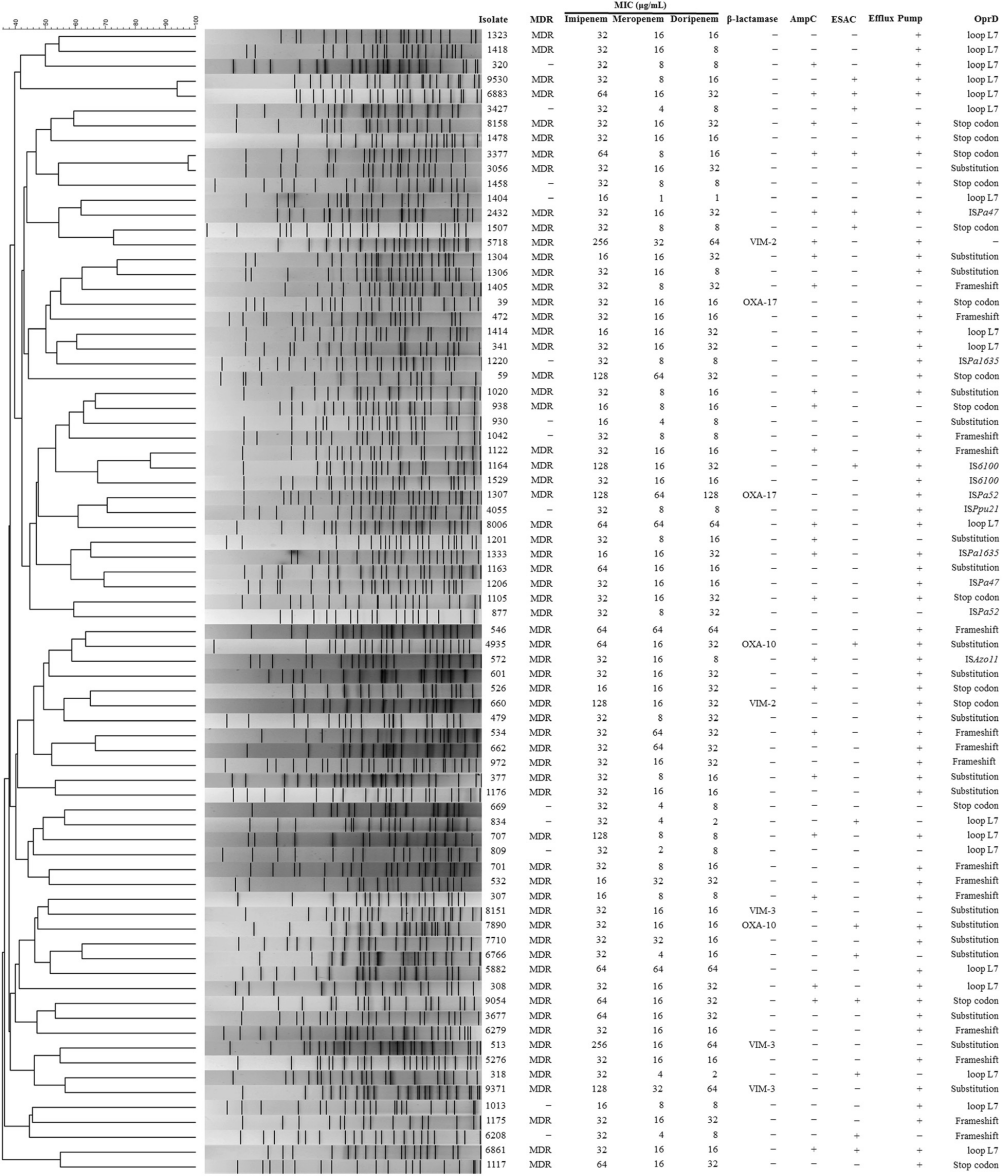
PFGE, MICs of carbapenems, and resistant mechanisms among 77 IRPA isolates. One isolate (926) showing low resolution of PFGE was excluded

PFGE was performed on all collected IRPA isolates except isolate 926 showed low resolution (Fig. 1). Using a > 80 % similarity cut-off point [36], PFGE analysis showed high genetic heterogeneity in 77 isolates. Only 3 pairs of isolates (9530 and 6883; 3377 and 3056; 1122 and 1164) collected from the same hospital and year showed genetic relatedness (Fig. 1).

### β-lactamase characterization

Genotyping of β-lactamases revealed that VIM-2, VIM-3, OXA-10, and OXA-17 β-lactamases were identified in 2 (2.6 %), 3 (3.8 %), 2 (2.6 %), and 1 (1.3 %) isolates, respectively. All VIM- and OXA-carrying strains were classified as MDR-*P. aeruginosa* (Fig. 1). AmpC overproduction and ESACs were shown in 25 (32.1 %) and 15 (19.2 %) of the 78 IRPA isolates, respectively (Fig. 1). The AmpC amino acid sequences of 15 ESACs producers showed 9 variants from the AmpC sequence of *P. aeruginosa* PAO1. The most frequent variant was PDC-3 (4/15, isolates 318, 1164, 6883, and 9530), which contained a T105A substitution, compared to the AmpC of *P. aeruginosa* PAO1 (PDC-1) [16]. The PDC-2 (1/15, isolate 7890) and PDC-5 (3/15, isolates 834, 6208, and 9054) variants had the G27D, A97V, T105A, and V205L; and R79Q and T105A substitutions, respectively. Only 2 isolates (2/15, isolates 1507 and 2432) showed the identical PAO1 sequence of AmpC. The remaining 5 isolates contained substitutions of AmpC described as follows: isolate 3377, G27D, T105A, A156T, V205L, and V356I; isolate 3427, R79Q, T105A, V205L, and V356I; isolate 4935, T21A, T105A, and V205L; isolate 6766, R79Q, T105A, and V205L; and isolate 6861, G27D, T105A, and V205.

### Efflux pump activity

A greater than or equal to fourfold decrease in MIC of meropenem when tested in combination with either efflux pump inhibitor, PAβN or CCCP, was shown in 58 (74.4 %) and 3 (3.8 %) of the IRPA isolates, respectively. This indicates that an active efflux pump contributes to the imipenem resistance of *P. aeruginosa* isolates. Moreover, PAβN was effective in changing the isolate categorization from meropenem resistant (MIC ≥ 8 μg/mL) to susceptible (MIC ≤ 2 μg/mL) in 40 isolates (40/58, 70 %). Overexpression of RND efflux pump genes *mexA*, *mexC*, *mexE*, and *mexX* were further determined among 29 selected isolates with greater than or equal to a fourfold concentration decrease in MIC (from resistant to susceptible) for meropenem in combination with PAβN. Among them, no overexpression of *mexA* was detected, and only 6 (20.7 %) of the isolates expressed *mexC* and *mexE* at least fivefold higher than that of the control strain PAO1. Eighteen (62.1 %) isolates overexpressed *mexX* at least tenfold higher than that of PAO1. Nine (31 %) of the 29 isolates that did not overexpress any of the major RND efflux pumps were further tested for the susceptibility to 50 μg/mL of PAβN, and the results showed 1 isolate (532) was susceptible to PAβN. However, the remaining 8 isolates possibly contained the additional PAβN-sensitive efflux pump that reduces the imipenem susceptibility.

### Outer membrane protein OprD analysis

The amino acid sequences of OprD from IRPA isolates were compared with the sequence of the reference strain PAO1. The results showed that *oprD* mutations with an amino acid substitution, shortened putative loop L7, a premature stop codon by point mutation, a frameshift by nucleotide insertion or deletion, or interruption by insertion sequence (IS) were found in 19 (24.4 %), 18 (23.1 %), 15 (19.2 %), 14 (17.9 %), and 10 (12.8 %) isolates, respectively. Only 2 isolates (2.6 %, isolates 926 and 5718) had the identical sequence of the PAO1 *oprD* (Table 2). Moreover, 7 IRPA isolates (669, 809, 877, 930, 1404, 3056, and 5276) contained only an *oprD* mutation but not other mechanisms associated with imipenem resistance (Fig. 1).

**Table 2:** *oprD* genotypes among 78 bacteremic IRPA isolates

Major types of mutation^a^	No. of isolate	Description
Amino acid frameshift produced by 1- and 2-bp insertion or deletion	10	Deletion of: 1 bp (G) at nt 311; 2 bp (TG) at nt 407-408; 1 bp (C) at nt 456 (2 isolates) Insertion of: 1 bp (G) at nt 555; 1 bp (T) at nt 977; 2 bp (AC) at nt 1050-1051 (2 isolates); 1 bp (C) at nt 1205 (2 isolates)
Amino acid frameshift produced by partial deletion	5	11-bp deletion beginning at nt 140, nt 175, nt 473 13-bp deletion beginning at nt 175, nt 208
Premature stop codon (point mutation)	14	CAG → TAG at nt 55-57, nt 472-474, nt 883-885, nt 1270-1272 (3 isolates) TGG → TAG at nt 193-195 TGG → TAG at nt 829-831 (3 isolates) TGG → TGA at nt 829-831 TGG → TAG at nt 1015-1017 (2 isolates) TAT → TAG at nt 1048-1050
Interruption by IS	10	Transposase at nt 37, 50 (2 isolates), 271, 434, 562, 571, 623, 652, 694
Amino acid substitution	19	Polymorphisms: D43N, S57E, S59R, T103S, K115T, V127L, F170L, E185Q, P186G, V189T, E202Q, I210A, E230K, D231N, S240T, L246V, D249E, L252V, N262T, R310E, A315G, S403A, Q424E, and G425A
Shortening of putative loop L7	18	Putative loop L7 (372 V-DSSSSYAGL-Y384)
Identical	2	-

^a^Only 2 isolates (926 and 5718) showed identical sequences of *oprD* to PAO1. The other 76 isolates contained amino acid variation compared to the sequence of *oprD* in PAO1

A shortened putative loop L7 of OprD was found in 18 isolates (23.1 %) (MIC ranges of imipenem, meropenem, and doripenem were 16-64, 1-64, and 1-64 μg/mL, respectively) (Fig. 1). Additive amino acid substitutions of OprD were found in all these 18 IRPA. In addition, isolates 472, 662, 6883, 8006, and 9530 contained identical 15 amino acid substitutions and a shortened loop L7 of OprD. Isolate 1404, with a shortened loop L7 alone, showed lower MICs of meropenem (1 μg/mL) and doripenem (1 μg/mL); the remaining 11 isolates had a shortened loop L7 in combination with AmpC and ESAC overproduction or active efflux overexpression and showed higher MICs of meropenem (4–64 μg/mL) and doripenem (2–32 μg/mL) (Fig. 1). The amino acid variation of isolate 1404 contributed to its low level resistance to imipenem (MIC, 16 μg/mL).

The sequencing results showed that 6 IS elements, including IS*Pa47* (isolates 1406 and 2432), IS*Pa1635* (isolates 1220 and 1333), IS*6110* (isolates 1164 and 1529), IS*Pa52* (isolates 877 and 1307), IS*Ppu21* (isolate 4055), and IS*Azo11* (isolate 572), were identified in 10 IRPA isolates, and thus lead to a disruption of the coding sequence of OprD (Fig. 1). Among these IS elements, IS*Pa47*, IS*Pa1635*, and IS*6110*, were classified as IS family IS*6330*, IS*4*, and IS*6*, respectively. IS*Pa52*, IS*Ppu21*, and IS*Azo11* were classified as IS*5* transposase family. No novel IS element was found in our IRPA isolates.

## Discussion

In the present study, we showed the prevalence of imipenem resistance in bacteremic *P. aeruginosa* is 5.9 % during a 10-year longitudinal surveillance in Taiwan. Mutations in *oprD* and an active efflux pump are the major causes of imipenem resistance, followed by overproduction of AmpC β-lactamase, ESACs phenotype, and the acquisition of VIM- and OXA-β-lactamases in *P. aeruginosa*.

Surveillance of antimicrobial susceptibility by the SMART 2008 reported that the susceptibility to imipenem of *P. aeruginosa* from intra-abdominal infections was 69 % worldwide [9]. Another global multicenter surveillance study, Tigecycline Evaluation and Surveillance Trial (TEST), showed that the imipenem resistance rate of blood isolates of *P. aeruginosa* collected from 2004 until August 2009 was 7.4 % [8]. Recently, carbapenem resistance of invasive *P. aeruginosa* has rising to 17.1 %, which was reported from the European Centre for Disease Prevention and Control 2012 [11]. Lin et al. showed the overall prevalence of CRPA was 10.2 % in Taiwan, and there was a significant trend of increasing CRPA prevalence during the period 2000-2010 [10]. However, our results showed lower prevalence (5.9 %) of bacteremic CRPA isolates in Taiwan. Moreover, the entire collection was highly susceptible to amikacin (67/78, 85.9 %) and no colistin resistant IRPA isolate was found. As a result, amikacin and colistin can serve as treatment agents against bacteremic IRPA in Taiwan. However, carbapenem susceptibility of invasive *P. aeruginosa* infections needs to be continually monitored to control the prevalence of CRPA.

The active efflux pumps in *P. aeruginosa* belonging to the RND family, which contributes to multidrug resistance by extruding the antimicrobial agents outside the bacterial cells [37]. Overexpression of the RND efflux pump systems MexAB-OprM, MexCD-OprJ, MexEF-OprN, and MexXY-OprM confer resistance to carbapenem in *P. aeruginosa* [2, 12, 13]. In this study, the *mexA* is constitutively expressed in all of the tested isolates with active efflux, and no difference among imipenem resistant isolates was found, compared with *P. aeruginosa* PAO1. This indicates that *mexA* contributes a limited role to imipenem resistance in our isolates. The *mexC*, *mexE*, and *mexX* are expressed at a low level in late log phase in PAO1, but their expression is more than fivefold up-regulated in 6 (20.7 %), 6 (20.7 %), and 18 (62.1 %) of 29 selected resistant strains, respectively. Therefore, our data demonstrated the importance of efflux pump systems in the imipenem resistance of *P. aeruginosa*. However, 8 isolates which possibly contained unknown PAβN-sensitive efflux pumps that reduce imipenem susceptibility remained to be investigated.

The outer membrane protein OprD, a carbapenem-specific porin, contributed to imipenem resistance and reduced susceptibility to meropenem in *P. aeruginosa* [5, 6, 38]. Mutations of *oprD* prevalent in our isolates revealed that loss or reduced OprD confers resistance to carbapenem in *P. aeruginosa* (Fig. 1). A shortened putative loop L7 of the OprD porin was identified in carbapenem-resistant isolates, and this shortening may open the porin channel to allow optimal penetration of meropenem and increase the meropenem susceptibility profile in imipenem-resistant *P. aeruginosa* [6, 38]. We also found a shortened loop L7 of the OprD in 18 IRPA isolates, and strain 1404, with only a shortened loop L7, showed lower MICs of meropenem (1 μg/mL) and doripenem (1 μg/mL), compared with strains had shortened loop L7 and the presence of AmpC and ESAC overproduction or active efflux (4–64 μg/mL). A lower MIC of doripenem in strains with a shortened loop L7 of OprD was also observed (Fig. 1). This raises the possibility that a shortened loop L7 of the OprD porin is responsible for the unusual meropenem and doripenem hypersusceptibility. Moreover, Riera et al. showed that while the inactivation of the porin OprD caused high-level resistance to imipenem (MIC > 32 μg/mL), it produced only moderate resistance to meropenem [7], and these results are observed in this study (Fig. 1).

Yan et al. have reported the presence of unusual *bla*-encoding integrons of VIM-2, VIM-3, OXA-10, and OXA-17 in multidrug-resistant *P. aeruginosa* isolates in Taiwan [28]. In the present study, all 8 VIM- and OXA-carrying isolates belonged to MDR *P. aeruginosa*. Although the imipenem resistance mechanisms in our isolates are diverse, isolates harboring VIM- and OXA-type β-lactamases showed higher carbapenem MICs, especially imipenem (32–256 μg/mL) (Fig. 1). This reflects that carbapenem can be efficiently hydrolyzed by the VIM- and OXA-type β-lactamases [5, 12].

Increased production of the AmpC chromosome-encoded cephalosporinase and ESACs production contributed to carbapenem resistance in *P. aeruginosa* [2, 5, 7, 13, 16]. In this study, we used the AmpC inhibitor cloxacillin to demonstrate that AmpC overproduction and ESAC were shown in 25 (32.1 %) and 15 (19.2 %) of the IRPA strains, respectively. The prevalence of the AmpC and ESAC phenotypes in our isolates was lower than the reported by Rodríguez-Martínez et al. (78 % and 72 %) [16], and we revealed that overproduction of AmpC and ESAC play an additive role in reduced susceptibility to carbapenem in our resistant strains. Moreover, 88.2 % (30/34) of AmpC or ESAC overproducers were MDR strains, indicating that overexpression of AmpC and ESAC is an important issue for antibiotic resistance in *P. aeruginosa*. Thirteen out of 15 isolates contained an AmpC β-lactamase variant, with all variants possessing an alanine residue at position 105. This residue had been previously shown to be the key factor for an ESAC phenotype [16]. However, 2 isolates (1507 and 2432) were ESAC producers containing the identical PAO1 (PDC-1) sequence of AmpC. As a result, the mechanism leading to the EASC property in these two isolates remained to be clarified.

## Conclusions

In conclusion, imipenem resistance in bacteremic *P. aeruginosa* collected between January 2000 and February 2010 remained low (5.9 %) in Taiwan. Mutations of *oprD* and active efflux are the major causes of imipenem resistance. Overproduction of AmpC β-lactamase, ESAC, and acquisition of VIM and OXA are also involved in imipenem resistance of *P. aeruginosa*. Since 83.3 % of imipenem-resistant isolates were found to be MDR strains, usage of carbapenems should be controlled to prevent pan-drug resistant *P. aeruginosa* occurrence.

## Abbreviations

CCCP, carbonyl-cyanide-*m*-chlorophenylhydrazone; CRPA, carbapenem-resistant *P. aeruginosa*; ESACs, extended-spectrum AmpC cephalosporinases; IS, insertion sequence; IRPA, imipenem-resistant *Pseudomonas aeruginosa*; MDR, multidrug resistant; MH, Mueller-Hinton; MIC, Minimal inhibitory concentration; PAβN, Phe-Arg β-naphthylamide dihydrochloride; RND, resistance-nodulation-division.

## Additional file

Additional file 1: Table S1. Oligonucleotide primers used in this study. (DOC 106 kb)
