# Supplementary material for: Overproduction of active efflux pump and variations of OprD dominate in imipenem-resistant Pseudomonas aeruginosa isolated from patients with bloodstream infections in Taiwan
Source: BMC Microbiol. 2016 Jun 13;16:107. doi: 10.1186/s12866-016-0719-2 (PMC4906909; doi:10.1186/s12866-016-0719-2)
Supplement: Additional file 1: Table S1. — Oligonucleotide primers used in this study. (DOC 106 kb) [file 12866_2016_719_MOESM1_ESM.doc]

Table S1. Oligonucleotide primers used in this study.

| Primer | Sequence (5’-3’) | Reference |
| --- | --- | --- |
| **β-lactamase amplification and sequencing** | |  |
| IMP-1 | TGAGCAAGTTATCTGTATTC | [1] |
|  | TTAGTTGCTTGGTTTTGATG |  |
| IMP-2 | GGCAGTCGCCCTAAAACAAA | [1] |
|  | TAGTTACTTGGCTGTGATGG |  |
| VIM-1 | TTATGGAGCAGCAACGATGT | [1] |
|  | CAAAAGTCCCGCTCCAACGA |  |
| VIM-2 | AAAGTTATGCCGCACTCACC | [1] |
|  | TGCAACTTCATGTTATGCCG |  |
| SPM | CCTACAATCTAACGGCGACC | [2] |
|  | TCGCCGTGTCCAGGTATAAC |  |
| KPC | GCTACACCTAGCTCCACCTTC | [3] |
|  | GCATGGATTACCAACCACTGT |  |
| GIM | AGAACCTTGACCGAACGCAG | [4] |
|  | ACTCATGACTCCTCACGAGG |  |
| SIM | TACAAGGGATTCGGCATCG | [5] |
|  | TAATGGCCTGTTCCCATGTG |  |
| PSE | GCTTTTAATACCATCCGTGG | [6] |
|  | AGCGCGACTGTGATGTATAA |  |
| CTX-M-13 | GGTTAAAAAATCACTGCGTC | [7] |
|  | TTGGTGACGATTTTAGCCGC |  |
| CTX-M-9 | ATGGTGACAAAGAGAGTGCA | [7] |
|  | CCCTTCGGCGATGATTCTC |  |
| TEM | CCCCTATTTGTTTATTTTTCT | [8] |
|  | GACAGTTACCAATGCTTAAT |  |
| SHV | CCGGGTTATTCTTATTTGTC | [9] |
|  | TAGCGTTGCCAGTGCTCGAT |  |
| GES | ATGCGCTTCATTCACGCAC | [10] |
|  | CTATTTGTCCGTGCTCAGGA |  |
| OXA-1 | TCCACAAACGCTGAAATTGCT | This study |
|  | CGACCCCAAGTTTCCTGTAAGT |  |
| OXA-2 | GGTCAGGCGCGAGGCTGTCT | This study |
|  | GGTCAGGCGCGAGGCTGTCT |  |
| OXA-23 | GATCGGATTGGAGAACCAGA | [11] |
|  | ATTTCTGACCGCATTTCCAT |  |
| OXA-40 | GTA CTA ATC AAA GTT GTG AA | This study |
|  | TTC CCC TAA CAT GAA TTT GT |  |
| OXA-48 | ATCAAGAGCTTGCAAGACGGAC | [12] |
|  | CAGTCAACCAACCTACCTGTGG |  |
| OXA-51 | TAATGCTTTGATCGGCCTTG | [11] |
|  | TGGATTGCACTTCATCTTGG |  |
| OXA-58 | AAGTATTGGGGCTTGTGCTG | [11] |
|  | CCCCTCTGCGCTCTACATAC |  |
| ***oprD*** **amplification and sequencing** | | |
| OprD-F | CGCCGACAAGAAGAACTAGC | [13] |
| OprD-R | GTCGATTACAGGATCGACAG |  |
| OprD-F2 | GCCGACCACCGTCAAATCG |  |
| ***ampC*** **amplification and sequencing** | | |
| PreAmpC-PA1 | ATGCAGCCAACGACAAAGG | [14] |
| PostAmpC-PA2 | CGCCCTCGCGAGCGCGCTTC |  |
| ampC-PA-A | CTTCCACACTGCTGTTCGCC | [14] |
| ampC-PA-B | TTGGCCAGGATCACCAGTCC- |  |
| **RT-qPCR** | | |
| proC | CAGGCCGGGCAGTTGCTGTC | [15] |
|  | GGTCAGGCGCGAGGCTGTCT |  |
| mexA | AACCCGAACAACGAGCTG | [16] |
|  | ATGGCCTTCTGCTTGACG |  |
| mexC | GGAAGAGCGACAGGAGGC | [16] |
|  | CTGCACCGTCAGGCCCTC |  |
| mexE | TACTGGTCCTGAGCGCCT | [16] |
|  | TCAGCGGTTGTTCGATGA |  |
| mexX | GGCTTGGTGGAAGACGTG | [16] |
|  | GGCTGATGATCCAGTCGC |  |

**References**

1. Yan JJ, Hsueh PR, Ko WC, Luh KT, Tsai SH, Wu HM, Wu JJ. Metallo-beta-lactamases in clinical *Pseudomonas* isolates in Taiwan and identification of VIM-3, a novel variant of the VIM-2 enzyme. Antimicrob Agents Chemother. 2001;45:2224–8.
2. Gales AC, Menezes LC, Silbert S, Sader HS. Dissemination in distinct Brazilian regions of an epidemic carbapenem-resistant *Pseudomonas aeruginosa* producing SPM metallo-beta-lactamase. J Antimicrob Chemother. 2003;52:699–702.
3. Smith Moland E, Hanson ND, Herrera VL, Black JA, Lockhart TJ, Hossain A, et al. Plasmid-mediated, carbapenem-hydrolysing beta-lactamase, KPC-2, in *Klebsiella pneumoniae* isolates. J Antimicrob Chemother. 2003;51:711–4.
4. Castanheira M, Toleman MA, Jones RN, Schmidt FJ, Walsh TR. Molecular characterization of a beta-lactamase gene, *bla*_GIM-1_, encoding a new subclass of metallo-beta-lactamase. Antimicrob Agents Chemother. 2004;48:4654–61.
5. Lee K, Yum JH, Yong D, Lee HM, Kim HD, Docquier JD, et al. Novel acquired metallo-beta-lactamase gene, bla(SIM-1), in a class 1 integron from *Acinetobacter baumannii* clinical isolates from Korea. Antimicrob Agents Chemother. 2005;49: 4485–91.
6. Yan JJ, Hsueh PR, Lu JJ, Chang FY, Ko WC, Wu JJ. Characterization of acquired beta-lactamases and their genetic support in multidrug-resistant *Pseudomonas aeruginosa* isolates in Taiwan: the prevalence of unusual integrons. J Antimicrob Chemother. 2006;58:530–6.
7. Saladin M, Cao VT, Lambert T, Donay JL, Herrmann JL, Ould-Hocine Z, et al. Diversity of CTX-M beta-lactamases and their promoter regions from Enterobacteriaceae isolated in three Parisian hospitals. FEMS Microbiol Lett 2002;209:161–8.
8. Yan JJ, Ko WC, Wu JJ. Identification of a plasmid encoding SHV-12, TEM-1, and a variant of IMP-2 metallo-beta-lactamase, IMP-8, from a clinical isolate of *Klebsiella pneumoniae*. Antimicrob Agents Chemother 2001;45:2368–71.
9. Nuesch-Inderbinen M T, Hachler H, Kayser FH. Detection of genes coding for extended-spectrum SHV beta-lactamases in clinical isolates by a molecular genetic method, and comparison with the E test. Eur J Clin Microbiol Infect Dis 1996;15:398–402.
10. Poirel L, Thomas ILe, Naas T, Karim A, Nordmann P. Biochemical sequence analyses of GES-1, a novel class A extended-spectrum beta-lactamase, and the class 1 integron In52 from *Klebsiella pneumoniae*. Antimicrob Agents Chemother 2000;44:622–32.
11. Woodford N, Ellington MJ, Coelho JM, Turton JF, Ward ME, Brown S, et al. Multiplex PCR for genes encoding prevalent OXA carbapenemases in *Acinetobacter* spp. Int J Antimicrob Agents 2006;27:351–3.
12. Poirel L, Heritier C, Tolun V, Nordmann P. Emergence of oxacillinase-mediated resistance to imipenem in *Klebsiella pneumoniae*. Antimicrob Agents Chemother 2004;48:15–22.
13. Rodriguez-Martinez JM, Poirel L, Nordmann P. Molecular epidemiology and mechanisms of carbapenem resistance in *Pseudomonas aeruginosa*. Antimicrob Agents Chemother. 2009;53:4783–8.
14. Rodriguez-Martinez JM, Poirel L, Nordmann P. Extended-spectrum cephalosporinases in *Pseudomonas aeruginosa*. Antimicrob Agents Chemother. 2009;53:1766–71.
15. Savli H, Karadenizli A, Kolayli F, Gundes S, Ozbek U, Vahaboglu H. Expression stability of six housekeeping genes: A proposal for resistance gene quantification studies of *Pseudomonas aeruginosa* by real-time quantitative RT-PCR. J Med Microbiol. 2003;52:403–8.
16. Quale J, Bratu S, Gupta J, Landman D. Interplay of efflux system, *ampC*, and *oprD* expression in carbapenem resistance of *Pseudomonas aeruginosa* clinical isolates. Antimicrob Agents Chemother. 2006;50:1633–41.
